# Magnetodynamics in exchange coupled composite magnets

**DOI:** 10.1038/s41598-025-04367-9

**Published:** 2025-07-01

**Authors:** Richa Bhardwaj, Antonio Caretta, Simone Laterza, Gabriele Coltrioli, Francesco Cautero, Amardeep Bharti, Kapil Dev, Pietro Parisse, Cinzia Di Giorgio, Subramanian Annapoorni, Asokan Kandasami, Marco Malvestuto

**Affiliations:** 1 Elettra Sincrotrone Trieste S.C.p.A., Strada Statale 14-km 163.5 in AREA Science Park, 34149 Basovizza, Trieste, Italy; 2 Department of Physics, University of Trieste, Via A. Valerio 2, 34127 Trieste, Italy; 3 Department of Physics and Astrophysics, University of Delhi, Delhi, 110007 India; 4 Istituto Officina dei Materiali (CNR-IOM), SS 14 km 163.5, 34149 Trieste, Italy; 5 Materials Foundry Institute, National Research Council (CNR-IOM), Trieste, Italy; 6 Department of Physics & Centre for Interdisciplinary Research, University of Petroleum and Energy Studies (UPES), Dehra Dun, 248007 India

**Keywords:** Magneto-optics, Kerr effect, Magnetization dynamics, ultrafast magnetic effects, Magnetic properties and materials, Spintronics, Magnetic properties and materials, Free-electron lasers, Optical spectroscopy

## Abstract

Tailoring magnetization using all-optical methods in exchange-coupled composite magnets holds significant scientific potential for advancing ultrafast magneto-optic devices. Here, we investigate the femtosecond laser-activated magnetization dynamics in FeNi/FePt composites using time-resolved magneto-optical Kerr effect (tr-MOKE) spectroscopy across the both visible and extreme ultraviolet spectral ranges. Observations from visible tr-MOKE reveal ultrafast demagnetization and an initial magnetization reorientation, characterized by a transient increase in the in-plane magnetization vector occurring on timescales shorter than 500 fs. Core-resonant tr-MOKE at Ni, Fe $\hbox {M}_{2,3}$ and Pt $\hbox {N}_{6,7}$-edge provide an element-specific contribution to the magnetodynamics. We infer that the sub picosecond magnetodynamics arises from the interplay between spin disordering and the dynamic nature of interlayer exchange coupling between FeNi and FePt in FeNi/FePt.

## Introduction

Within the realm of magnetism and materials science, exchange-coupled composite (ECC) magnets have a significant impact on the advancements and explorations in the field of spintronics due to their ability to integrate individual materials with distinct magnetic properties^1–3^. Coupling of soft and hard magnetic phases in perpendicular ECC exhibits exceptional thermal stability and reduced switching fields and thus shows tremendous prospects for ultrahigh density magnetic recording, magnetic sensors, quantum computing, and magnonic devices^4–6^.

In ECC magnets, the critical role is played by the interfacial exchange coupling between the soft and hard magnetic phases, as it significantly influences the magnetization dynamics by modulating the magnetic interactions between the two layers. Notably, it has been observed that the intermixing/diffusion in the vicinity of the interface can intensify the exchange coupling strength, as observed in the SmCo/Fe(Co) system^7^. Additionally, the application of laser excitation also demonstrates an influence on the dynamic nature of the coupling strength, as an example FePt/CoFe and FePt/FeNi^8,9^. These investigations shed light on the complex interplay among interfacial exchange coupling, laser excitation, and magnetic properties.

The intriguing findings of laser-induced spin dynamics in ECC such as FePt/CoFe^10^, and TbFeCo/GdFeCo^11^ employ the classical Landau-Lifshitz-Gilbert (LLG) equation to detect the spin precession and damping behavior. Nevertheless, to fully exploit the benefits of ECC, it is important to understand the magnetization dynamics on femtosecond timescales, where the classical LLG approximation breaks down since the modulus of the magnetization is not conserved during the overall process.

In this study, the focus lies on examining the ultrafast optically induced magnetization dynamics in the FeNi/FePt composite system. The choice of the FeNi alloy as the soft magnetic component is based on its high saturation magnetization, while the selection of FePt as the hard magnetic layer is attributed to its robust perpendicular anisotropy, which can be flexibly tuned across a broad range owing to its magnetocrystalline anisotropy constant^12^. The examination of the modulated magnetization dynamics in FeNi/FePt composite system assumes critical importance for the practical implementation of devices.

Our work employs time-resolved magneto-optical Kerr effect (tr-MOKE) spectroscopy to analyze the film’s magnetodynamics in the visible range. Further, to retrieve information about individual element contribution to magnetization and inter-site spin interaction, free electron laser(FEL)-based core-resonant tr-MOKE was performed by being sensitive to the M-edge of Ni and Fe, and N-edge of Pt. Our investigations reveal a fast magnetization reorientation as a transient increase in the in-plane component of the magnetization vector on sub-picosecond timescales in the visible tr-MOKE, triggered by the intense laser pulse in ECC magnets. Notably, optically induced demagnetization can be leveraged to trigger the precessional motion of magnetization around the effective magnetic field. For instance, previous studies have reported coherent magnetization rotation induced by optical methods in exchange-coupled NiFe/NiO bilayers^13^. This behavior has also been observed in ferromagnet/semiconductor layered structures, such as MnGa/GaAs^14^ and EuO/Co^15^, where the observed increase in magnetization was attributed to the superdiffusive spin transport mechanism.

**Fig. 1:**
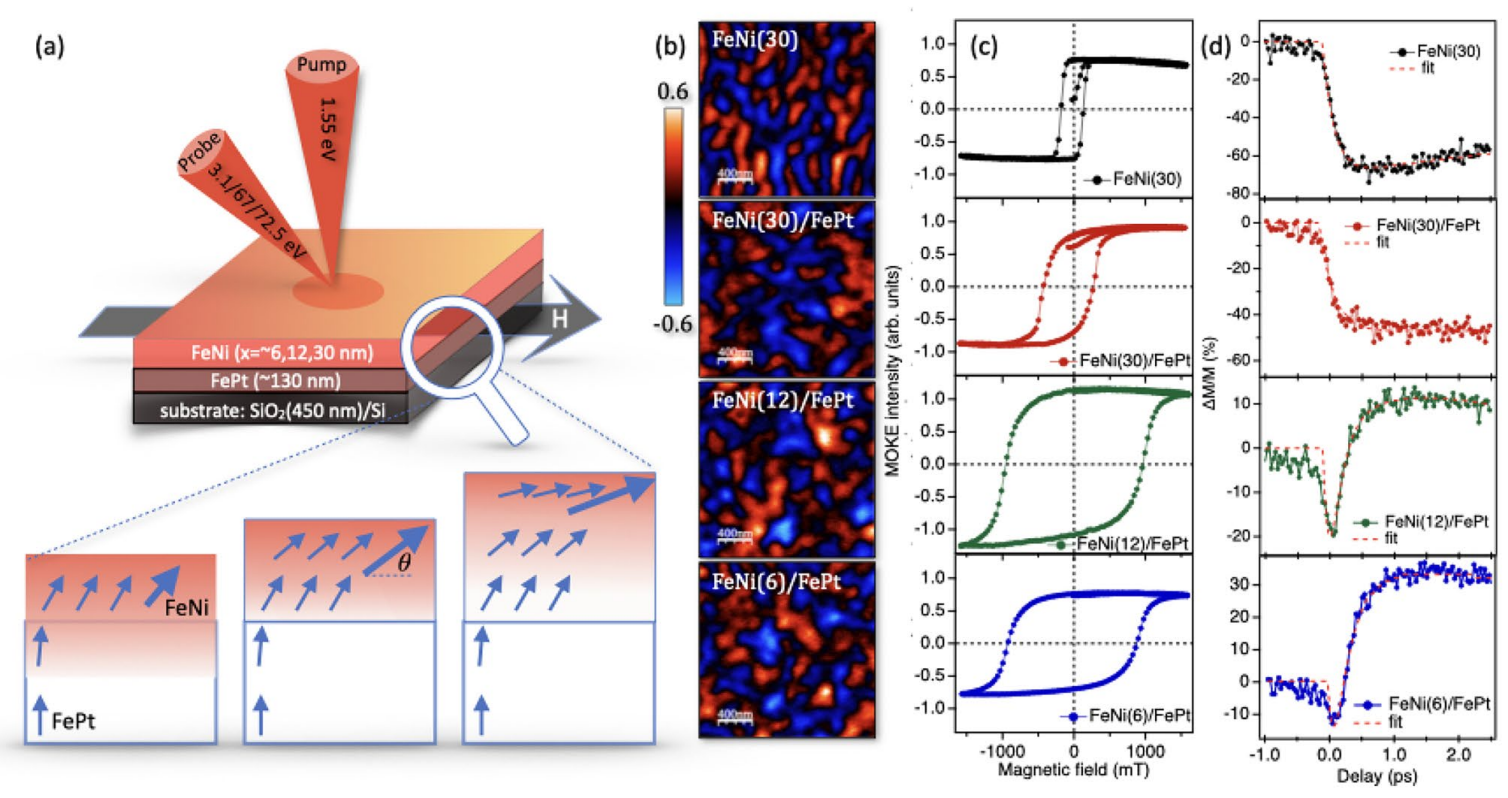
(**a**) Schematic of tr-MOKE experiment: A 1.55 eV pump pulse excites the sample, followed by probing at different photon energies-3.1 eV in the visible range and 67 eV and 72.5 eV in the extreme ultraviolet (EUV) range. The inset depicts the FeNi/FePt bilayer system with varying FeNi thicknesses. Blue arrows indicate the magnetization easy axis for FeNi and FePt. The red-shaded region highlights the pump-probe penetration depth, while the bold blue arrow represents the average magnetization canting detected by the probe pulse. (**b**) MFM phase maps: Magnetic force microscopy (MFM) images (2 ${\upmu }$m $\times$ 2 ${\upmu }$m) display the FeNi/FePt system with FeNi thicknesses ranging from 30 nm to 6 nm. All maps use a consistent color contrast for direct comparison. Notably, FeNi(12)/FePt and FeNi(6)/FePt exhibit a higher density of bright regions due to enhanced out-of-plane magnetization. (**c**) Static longitudinal MOKE measurements: Magneto-optical Kerr effect (MOKE) data for FeNi and FeNi/FePt composites show variations in magnetization as a function of FeNi thickness. (**d**) Magnetization dynamics: Time-resolved magnetization measurements reveal ultrafast demagnetization followed by recovery. FeNi(30) and FeNi(30)/FePt exhibit slower recovery dynamics, while FeNi(6)/FePt and FeNi(12)/FePt show ultrafast demagnetization accompanied by a transient rotation of magnetization vector with an increase of its in-plane component on femtosecond timescales before recovery.

For this work, thin films with element composition $\hbox {Fe}_{{20}}$ $\hbox {Ni}_{{80}}$(30nm) and $\hbox {Fe}_{{20}}$ $\hbox {Ni}_{{80}}$[x]/$\hbox {Fe}_{{46}}$ $\hbox {Pt}_{{54}}$[$\sim$130 nm], x= 6, 12, 30 nm, were deposited over $\hbox {SiO}_2$/Si substrate using two target DC/RF magnetron sputtering. Commercially purchased $\hbox {Fe}_{{46}}$ $\hbox {Pt}_{{54}}$ and $\hbox {Fe}_{{20}}$ $\hbox {Ni}_{{80}}$ alloy targets of 99.9% purity were used. Before deposition, the vacuum in the sputtering chamber was at 4$\times$ $10^{-6}$ mBar. Before deposition, the $\hbox {SiO}_2$/Si substrates were thoroughly cleaned by dipping them in piranha solution for 10 min. Subsequently, these substrates were rinsed in deionized water for 10 min after ultrasonication. For deposition, the pressure of the inert Argon gas in the deposition chamber was maintained at 3$\times$ $10^{-2}$ mBar. Before deposition, the targets were cleaned by initial rough sputtering (sputtering without substrates) for 15 min. The bottom layer of FePt was deposited at 15 W DC power for 15 minutes and the rotation of the substrate holder was maintained at 40 rpm. In addition, a FeNi top layer was deposited using a high RF power of 180 W. The layer thickness was controlled by varying the deposition time. Later, as-deposited films were annealed in a microprocessor controlled tubular furnace at $500^\circ$C for 5 h in the presence of Ar(95%) +H2(5%) gas mixture to reduce surface oxidation^16^. Magnetic force microscopy (MFM) measurements were performed to quantitatively compare the strength of out-of-plane magnetization component and domain size. MFM images were captured using an MFP-3D instrument (Oxford Instruments-Asylum Research) in nap mode, employing an HQ:NSC18/Co-Cr/AlBS cantilever (Mikromasch) with a radius of curvature < 60 nm and a spring constant of 2.8 N/m. Prior to imaging, the tip was magnetized vertically using a permanent magnet. The nap-mode distance was consistently maintained at 100 nm across all samples. Vibrating Sample Magnetometry (VSM) measurements were carried out (VSM, Microsense EV-9) on FeNi/FePt bilayer structure (Supplementary document, section III). The hysteresis curves clearly indicate that as the thickness of the top FeNi layer decreases from 30 nm down to 6 nm, the easy axis of magnetization undergoes a transformation from in-plane towards out-of-plane alignment. This observed transition is further corroborated by MFM results. It is to be noted that, the Rutherford backscattering spectrometry (RBS) performed on these samples reveals the presence of a diffused region at the FeNi/FePt interface. The estimated thickness and interlayer atomic diffusion values from RBS are given in the supplementary section IV.

Figure 1a illustrates the spin orientation in FeNi and FePt layers in FeNi/FePt composite system and the tr-MOKE experiment configuration. The top FeNi layer, which has an in-plane easy axis of magnetization, is canted by the buried FePt, which has an out-of-plane magnetization vector. Upon segmenting the FeNi film into horizontal layers, the magnetic moment’s canting within each layer is influenced by its proximity to the interface. This phenomenon arises from the interplay between shape anisotropy energy within the layer and the exchange interaction with the FePt layer, which possesses perpendicular magnetization. As the distance from the interface increases, the canting of magnetization vectors within the FeNi diminishes gradually, ultimately aligning parallel to the interface. Moreover, it is essential to consider the penetration depth of light in these measurements, which is approximately 13 nm. The estimate of the optical penetration depth, where the light has fallen to 1/e is around $\lambda$/4$\pi$k, where k is imaginary part of the refractive index of material whose value is taken from references^17,18^. Additionaly, MFM images (2 $\mu$m $\times$ 2 $\mu$m) display the FeNi/FePt system with increasing FeNi thicknesses. As highlighted in Fig. 1b, FeNi(12)/FePt and FeNi(6)/FePt exhibit a higher density of bright regions due to enhanced out-of-plane magnetization. Static MOKE measurements were performed in longitudinal geometry, sensitive to an in-plane component of magnetization. The interaction between the gradient of magnetization vector canting, influenced by proximity to the interface, and the optical probe’s sensitivity due to limited penetration depth, leads to thickness-dependent hysteresis, illustrated in Fig. 1c. This demonstrates the correlation between the measured in-plane magnetic component and the thickness of the film, emphasizing the intricate relationship between magnetic interactions and structural dimensions in layered magnetic systems. The magnetization dynamics were studied by pump-probe means in the visible and extreme ultraviolet (EUV) range.

**Visible pump-probe:** Longitudinal tr-MOKE measurements were performed in pump-probe mode using a Ti: Sapphire regenerative amplifier system that delivers intense pump pulses at 1.55 eV with a duration of $\sim$120 fs and a repetition rate of 1kHz. The schematic experimental geometry is shown in Fig. 1b. Samples were excited with a pump fluence of 19 mJ/$\hbox {cm}^2$. A frequency-doubling beta barium borate (BBO) crystal was utilized to generate a 3.1 eV probe pulse from the 1.55 eV source. The incident angle of probe pulse for was $30^\circ$. A Wollaston prism and a balanced photodiode scheme were employed for tr-MOKE detection and analysis.

**Core-resonant EUV pump-probe:** Core-resonant tr-MOKE measurements were carried out at the MagneDyn end-station, located at the externally seeded Extreme Ultraviolet Free-Electron Laser (EUV FEL) FERMI at Elettra Sincrotrone Trieste, Italy^19^. The sample was subjected to excitation by a $\sim$70 fs pump pulse at 1.55 eV with a 25 Hz repetition rate decimated with respect to the FEL 50 Hz repetition rate that allowed it to achieve the standard pump-on/off data acquisition mode. The pump pulse was generated by the same laser used to seed the FERMI FEL and exhibited a root mean square timing jitter regarding the FEL pulses of $\sim$7 fs. The probe consisted of linearly polarized $\sim$50 fs FEL light pulses whose energy is tuned to the absorption edge of Ni $\hbox {M}_{2,3}$ (67 eV), Fe $\hbox {M}_{2,3}$ (53.9 eV) and Pt $\hbox {N}_{6,7}$ (72.5 eV). The angle of incidence of the incoming probe pulse is $45^\circ$ and the pump and probe pulses are in a quasi-collinear configuration. The pump pulse at the sample was set at a fluence of 26 mJ/$\hbox {cm}^2$. The core-resonant tr-MOKE detection and analysis of the FEL light polarization angle $\theta _k$ as a function of the pump-probe time delay is carried out with a Wollaston-like polarimeter^20^.

The pump-probe measurements in visible and EUV ranges were conducted at room temperature with an applied magnetic field of 1.6 T. It is further important to note that fluence in both cases is defined as the energy density at which the intensity of the pulse drops to 1/$\hbox {e}^2$ of its maximum value^21^. The penetration depth of laser is $\sim$ 13 nm.

The Kerr rotation in both cases ($\theta _{k}$) can be approximated from the intensities as

1 $$\begin{aligned} \theta _{k} = \frac{1}{2} \frac{I_{{1}} - I_{{2}}}{I_{{1}} + I_{{2}}} \, \end{aligned}$$

where $I_1$ and $I_2$ are the intensities of two orthogonal components, which are used to approximate Kerr rotation. Finally, the magnetization dynamics are reconstructed from the observed Kerr rotation as

2 $$\begin{aligned} \frac{\Delta M}{M} (t) = \frac{\theta _k^{+} (t) - \theta _k^{-} (t)}{\theta ^{+}_{k(sat)} - \theta ^{-}_{k(sat)}} \, \end{aligned}$$

where t is the time delay between pump and probe arrival, $\theta _{k}^{+-}$ represents the MOKE signal, and $\theta _{k(sat)}^{+-}$ is the unpumped saturation values at opposite magnetic fields.

## Results

In Fig. 1c, the static MOKE for FeNi(30) and various FeNi/FePt composite samples with varying thicknesses of FeNi is shown. A clear trend is observed in the coercivity values, which decrease with increasing film thickness. This trend is attributed to the interlayer exchange coupling between the magnetically soft layer with high saturation (FeNi) and the neighboring layer (FePt) with a hard magnetic phase and high coercivity, resulting in an augmented maximum energy product for the composite system. This augmentation is particularly prominent for the thinner film, such as FeNi(6)/FePt. With the increase in the thickness of the top layer, the interlayer exchange coupling diminishes because the more distant layers from the interface are less affected by this interaction. Consequently, the hysteresis narrows and tends towards being governed primarily by shape anisotropy, as illustrated in the FeNi(30) sample (top panel of Fig. 1c). Figure 1d shows the relative change in magnetization characterized by tr-MOKE in the visible regime. The FeNi(30) sample (taken as a reference) undergoes an ultrafast demagnetization quenched to 65% and a slower recovery period. Similarly, FeNi(30)/FePt demonstrates a comparable behavior, with a different $\Delta {M/M}$ indicating a demagnetization of roughly 40%. The variation in the trace slope above 1 ps can be attributed to the fact that the recovery time significantly depends on how the system thermally dissipates energy received from the optical pulse. The effectiveness of energy transfer to the lattice and thermal energy transport depends on several factors, such as the substrate’s nature and the thermal coupling efficacy between various stacks of the system.

**Fig. 2:**
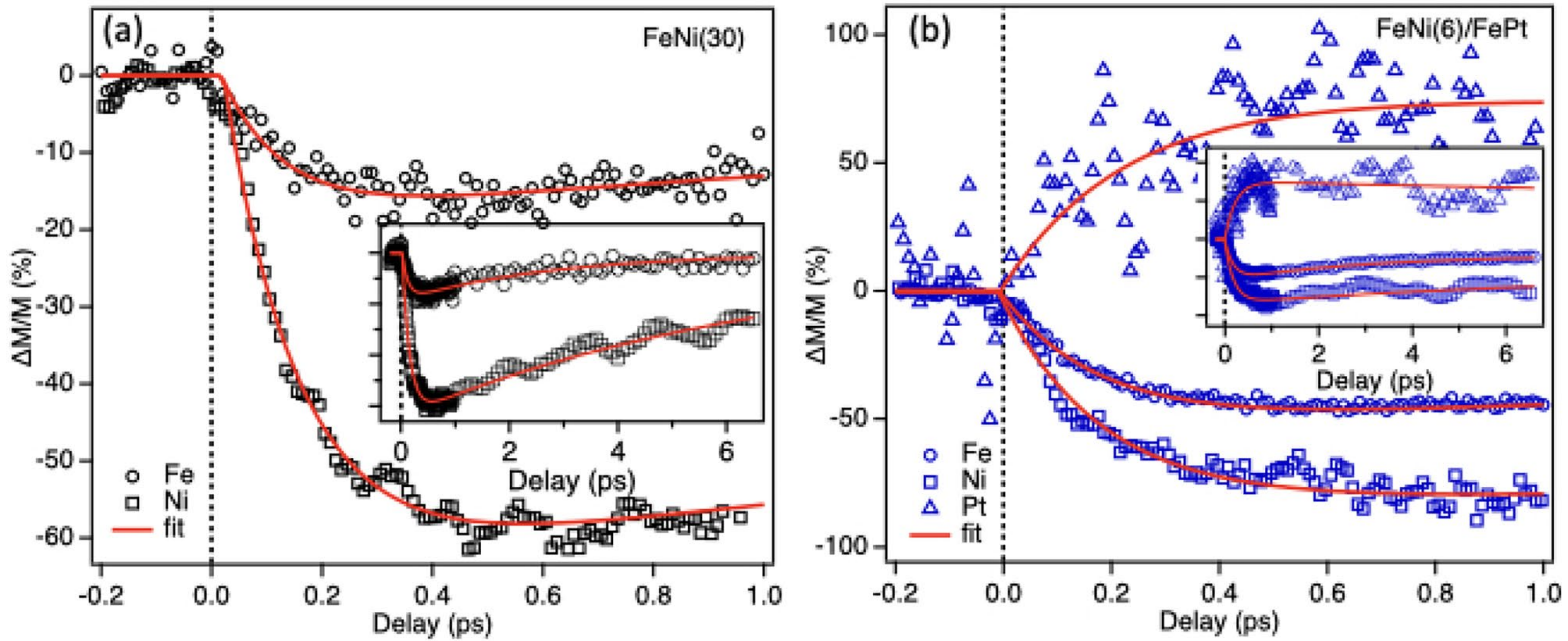
Relative change in magnetization dynamics sensitive to Ni, Fe $\hbox {M}_{2,3}$ and Pt $\hbox {N}_{6,7}$ edge for (**a**) FeNi(30) and (**b**) FeNi(6)/FePt. The inset highlights the extended recovery time, indicating prolonged relaxation processes in the thicker FeNi layer.

Interestingly, upon closer examination of the FeNi(12)/FePt and FeNi(6)/FePt samples, a clear pattern emerges, distinguishing them from the other samples. They exhibit an ultrafast demagnetization of the order of 18% and 14%, an increase of the transient longitudinal MOKE signal of 10% and 30%, that can be interpreted as an in-plane magnetization orientation. This increase refers to the comparison with the static MOKE initial magnetization, given in the supplementary. What is noteworthy is the short timescale of less than 500 fs within which the behavior is observed.

Further, to understand the behavior of composite samples showing contrasting features and to discern the contributions of each element to magnetodynamics, a core resonant tr-MOKE experiment were carried out on FeNi(30) and FeNi(6)/FePt. Figure 2 shows the magnetodynamics acquired at Ni, Fe $\hbox {M}_{2,3}$ and Pt $\hbox {N}_{6,7}$ edge. The Ni and Fe undergoes ultrafast demagnetization on a time scale of 200 fs and recover slowly. It’s worth noting that the delay trace at the Fe edge for FeNi(6)/FePt reflects non-distinguishing magnetization dynamics for both the top and bottom layers due to the probe’s penetration depth, which accesses both layers. On the other hand, the magnetization dynamics at the Pt N-edge show a contrasting behavior compared to the dynamics at the Ni and Fe. The signal-to-noise ratio of the time-resolved trace related to Pt’s magnetic dynamics is inferior compared to those of Fe and Ni, attributed to Pt’s significantly smaller yield at the N-edge. The unexpected positive shift in magnetic dynamics prompts further experimental investigation beyond this article’s scope, although a qualitative interpretation will be provided in the “Discussion” section.

The magnetization lifetimes were retrieved using a phenomenological decay-recovery exponential function, as previously described in the literature^22^. However, in the current scenario, the data exhibits distinct recovery times, namely an enhanced magnetization timescale for the visible tr-MOKE trace and a fast recovery for the resonant tr-MOKE trace, followed by a slower recovery in both cases. Therefore, the data was fitted with a function that contains an additional recovery term and is given by (Eq. 3).

3 $$\begin{aligned} f(t)= \frac{\Delta M}{M} \Theta (t)\Big (1 - e^{-t/\tau _{dm}} \Big )\ \Big (ie^{-t/\tau _{rec1}} + je^{-t/\tau _{rec2}}\Big ) \,, \end{aligned}$$

where $\Theta (t)$ is the Heaviside step function, *i*, *j* are the exponential amplitude constant, $\tau _{dm}$ is the characteristic time of initial ultrafast demagnetization, $\tau _{rec1}$ is the additional term corresponding to the transient enhanced magnetization for visible the tr-MOKE curve or fast recovery for the resonant tr-MOKE curve, and $\tau _{rec2}$ is associated with the slower thermal recovery towards initial equilibrium state, respectively. The lifetime values are listed in Tables 1 and 2.

**Table 1:** Demagnetization ($\tau _{dm}$) and recovery ($\tau _{rec1}$, $\tau _{rec2}$ time values extracted from the fitting of data in Fig. 1d.

Sample	$\tau _{dm}$ (fs)	$\tau _{rec1}(fs)$	$\tau _{rec2}$ (ps)
FeNi(30)	174 ± 13	–	14 ± 1 ps
FeNi(6)/FePt	140 ± 16	402 ± 40	17 ± 2
FeNi(12)/FePt	153 ± 16	529 ± 120	7 ± 1
FeNi(30)/FePt	213 ± 12	–	–

**Table 2:** Demagnetization ($\tau _{dm}$) and recovery ($\tau _{rec1}$, $\tau _{rec2}$) time values extracted from the fitting of data in Fig. 2.

Sample	Element	$\tau _{dm}$ (fs)	$\tau _{rec1} (ps)$	$\tau _{rec2}$ (ps)
FeNi(30)	Ni	157 ± 5	2.5 ± 0.2	20.5 ± 1.5
	Fe	170 ± 15	0.9 ± 0.4	4.5 ± 0.7
FeNi(6)/FePt	Ni	198 ± 11	9 ± 1.5	35 ± 3
	Fe	192 ± 5	2.4 ± 0.1	26 ± 3
	Pt	191 ± 45	–	–

**Fig. 3:**
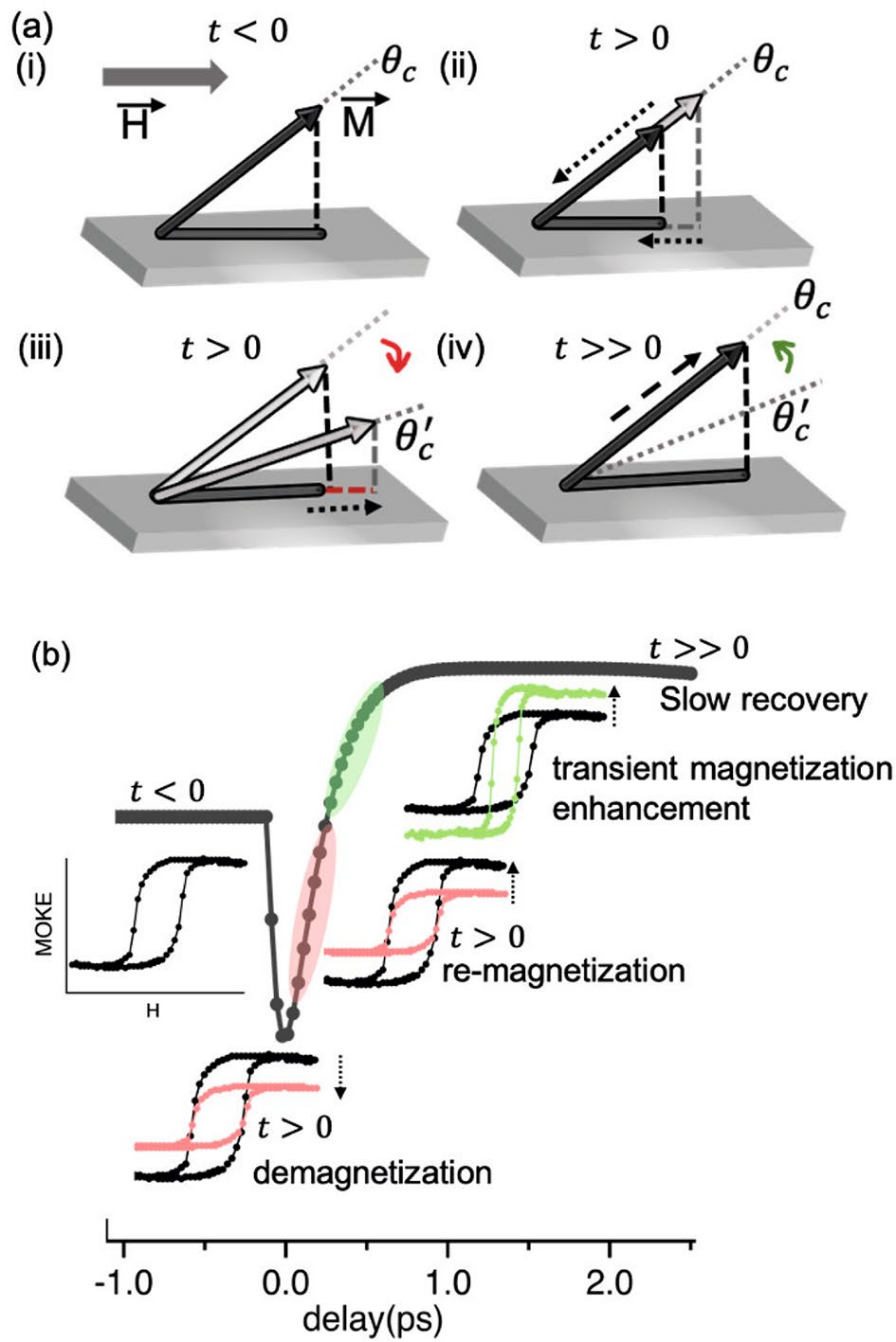
(**a**) Illustration of the observed magnetization dynamics: (i) For t<0, the net canted magnetization $\overrightarrow{M}$, $\theta _c$. $\overrightarrow{M}$ points in the equilibrium direction (grey dotted line). (ii) At t>0, $\overrightarrow{M}$ magnitude reduces, and (iii) a new equilibrium orientation,$\theta _c$ $^{\prime }$, due to altered exchange coupling, results in increased in-plane projection. A red dashed line projection shows the increase. The competition between processes (ii) and (iii) results in ultrafast demagnetization followed by an increase of the in-plane component of the magnetization vector. (iv) For t>1ps, the heat begins to dissipate, leading to the restoration of the magnitude of $\overrightarrow{M}$. (**b**) Illustration of the magnetization dynamics: static MOKE curve is quenched at t>0 (black to pecah color). Since at this point, the coupling strength is affected and considering the laser penetration depth, FeNi/FePt recovers by following the hysteresis of FeNi with higher MOKE rotation (green color) and later recovers to its original state on the longer timescale of ps.

## Discussion

The dynamics observed in visible tr-MOKE for FeNi(30) have been extensively elucidated in literature, taking into account the interplay of spin interactions and the impact of quantum fluctuations on magnetization dynamics. The demagnetization time scale = 174±13 fs is in accordance with literature within error limits^22,23^. $\tau _{dm}$ of FeNi(30)/FePt exhibits similar behavior to FeNi(30). On the other hand, for FeNi(6)/FePt and FeNi(12)/FePt, the magnetization dynamics show a transient rotation magnetization vector with an increase of its in-plane component rapidly after demagnetization on a sub-ps timescale, as shown in Fig. 1d. Similar short timescales for changes in the magnetization projection have been observed in previous studies^24,25^, demonstrating that rapid movement of the magnetization vector can potentially arise from effective fields. From this viewpoint, the modification of the interlayer exchange coupling can be interpreted as the transient modulation of an effective field term. A comparable behavior of about 10% ultrafast magnetic transient enhancement under given experiment conditions and at time scales of less than 1 ps is reported in MnGa/GaAs (ferromagnet/semiconductor) layered structures as a result of superdiffusive spin transports^14^. And on a time scale of 100 ps in EuO/Co bilayer due to photo-enhanced interfacial magnetic interactions^15^. Vomir et al.^24^ reported observing the initial change in both the modulus and orientation of the magnetization in a cobalt film deposited on sapphire, occurring within a few hundred femtoseconds.

The behavior observed in the current ECC FeNi/FePt magnet can be elucidated as follows:

1. The observed behavior can be effectively described by the mechanism illustrated in Fig. 3a. In the static scenario, the spins of the top layer exhibit canting due to the magnetic interlayer exchange coupling. The resultant canting from both layers, when projected in-plane, is depicted in Fig. 3a(i). When exposed to sudden heating by the pump pulse, two possible processes occur. Firstly, the magnitude of the magnetization, $\overrightarrow{M}$ , decreases as shown in Fig. 3a(ii). Secondly, the coupling strength diminishes, leading to a new equilibrium orientation, $\theta _c$ $^{\prime }$. This change results in a relative increase to the in-plane projection of the magnetization, as illustrated in Fig. 3a(iii). These two processes are believed to occur concurrently, which results in an ultrafast demagnetization followed by a transient increase in the in-plane component of the magnetization vector before eventually stabilizing, as depicted in Fig. 3a(iv).
2. Taking into account the micromagnetic presentation for ECC systems which involves the effective exchange coupling between soft and hard magnetic layers, we define the canted angle in each layer (angle of the magnetic moment with respect to the normal to the sample plane) as $\theta ^s$, for FeNi and $\theta ^h$ for FePt. The model allows us to deduce the ratio between these canted angles, which can be described as follows

4 $$\begin{aligned} r_\theta = \frac{\theta ^s}{\theta ^h} = \frac{cosh\left( L^h \sqrt{\frac{K^hb}{J^h}} \right) }{cos\left( L^s\sqrt{\frac{K^sa}{J^s}} \right) } \, \end{aligned}$$

where the superscripts *s* and *h* correspond to the soft and hard layers, respectively. *a* and *b* have a dependence on saturation magnetization ($M_s$) as described in detail in reference^26^. *L*, *K*, and *J* are the layer thickness, the magneto-crystalline anisotropy, and the exchange coupling parameter, respectively. Thus, change in magnetization after laser excitation can be expressed as

5 $$\begin{aligned} \frac{\Delta M}{M} \propto \frac{\theta _t^s/\theta _{sat}^s}{\theta _t^h/\theta _{sat}^h} -1 \,, \end{aligned}$$

where $\theta _t^s$ and $\theta _t^h$ are the canting angles in time after pump arrival and $\theta _{sat}^s$ and $\theta _{sat}^h$ before the pump arrival. Since FePt exhibits out-of-plane magnetization and in our MOKE configuration we are sensitive to the magnetization component parallel to the film surface, thus we assume that the change in magnetization in FePt is minute such that $\theta _t^h \approx \theta _{sat}^h$. Therefore Eq. (5) can be approximated to $\Delta M/M$ $\propto$ $\theta _t^s/\theta _{sat}^s$ – 1, which implies that change in magnetization mainly depends upon the change in $M_s$ and $J^s$ value in time of FeNi. The static value of $J^s$ for FeNi is of the order of 1$\times$ $10^{-6}$erg/cm which is modulated by $J^h$ of FePt with an approximate value of the same order^27^ and with incident pump fluence, respectively. The phenomenological model emphasizes that the observed transient increase in the in-plane component of the magnetization vector primarily stems from FeNi. This rotation is significantly influenced by FePt through the interfacial exchange coupling. Upon exposure to a pump, the interlayer coupling strength in the proximity of the interface experiences attenuation, leading the FeNi/FePt composite system to exhibit characteristics similar to pure FeNi. This leads to a positive variation of the MOKE rotation and a transient increase in the in-plane component of the magnetization vector. However, it eventually returns to its original state, as depicted in Fig. 3b. The unpumped $\theta _k$ for FeNi(30) is 0.7 mrad while for FeNi(6)/FePt and FeNi(12)/FePt are 0.35 mrad and 0.4 mrad.

Additionally, the results from the element-specific tr-MOKE show distinct dynamics at the Pt N-edge compared to the Ni and Fe M-edge. The underlying mechanism that governs the alignment of Pt spins, leading to magnetization at the Pt edge in FeNi(6)/FePt upon laser heating, remains a challenging puzzle that warrants further investigation and discussion. The EUV-MOKE measurements were conducted at an incidence angle of $45^\circ$ relative to the optical ones, due to experimental constraints of the MagneDyn beamline. This setup renders the EUV-MOKE sensitive to both in-plane and out-of-plane magnetization dynamics, contingent upon the initial magnetization state of the system. We propose two alternative qualitative scenarios to elucidate the observations. In the first scenario, the EUV probe, resonant to Pt, captures the dynamics of layers in proximity to the interface. Despite FePt being a hard magnet, FePt layers near the interface may experience influence from interlayer exchange coupling, leading to a canted average magnetization. Following the pump pulse and subsequent reduction in interlayer exchange coupling, the magnetization of the bottom layer returns to a fully out-of-plane orientation. The second scenario suggests that the Pt dynamics partially result from spin injection from the top layer to the bottom layer. Both scenarios will be the subject of dedicated experimental investigations. It is also essential to recognize the role of non-uniform atom diffusion, especially involving Pt, Ni and Fe, near the interface which can modify the exchange coupling.

In the end, the demagnetisation time scales observed for each element in EUV MOKE are consistent with those observed in visible MOKE within the error limit. Further, the positive sign of $\Delta M/M$ for Pt and the negative sign for Fe and Ni align well with the visible MOKE dynamics. Thus, the results obtained from element-specific MOKE complement the visible pump-probe MOKE dynamics, further validating our findings.

In conclusion, the present work explores the ultrafast magnetodynamics of the FeNi/FePt composite system by pump-probe tr-MOKE measurements. For small thicknesses of the FeNi layer in FeNi/FePt composites, a transient increase of approximately 30% in the in-plane component of the magnetization vector was observed within less than 500 fs, following an initial ultrafast demagnetization. We discuss the possible mechanisms of the interplay between spin disordering and the dynamic nature of exchange coupling between FeNi/FePt upon laser excitation. The results of resonant tr-MOKE at Pt and Ni edges remain challenging to explain and require further investigations. This work exhibits potential applications of soft/hard magnetic composites in spintronics and opens up new possibilities for manipulating magnetic properties

## Supplementary Information

Supplementary Information.

## Data Availability

The datasets used and/or analysed during the current study available from the corresponding author on reasonable request.
