# Supplementary material for: Magnetodynamics in exchange coupled composite magnets
Source: Sci Rep. 2025 Jul 1;15:21155. doi: 10.1038/s41598-025-04367-9 (PMC12218809; doi:10.1038/s41598-025-04367-9)
Supplement: Supplementary file 1 — Supplementary Information. [file 41598_2025_4367_MOESM1_ESM.pdf]

Magnetodynamics in exchange coupled composite magnets

I. Static Magneto-optical Kerr effect (MOKE): The static MOKE results for various FeNi/FePt samples in longitudinal geometry. The Kerr rotation is computed using equation (Eq. 1) in the main manuscript. In Table S1, Kerr rotation corresponding to each FeNi/FePt sample is tabulated.

TABLE S1: Kerr rotation measured from static MOKE hysteresis curve on FeNi/FePt samples.

**Sample**	**Kerr rotation (mrad)**
FeNi(30)	0.70
FeNi(6)/FePt	0.35
FeNi(12)/FePt	0.40
FeNi(30)/FePt	0.60

II Magnetic force microscopy (MFM): Fig. S1 shows the MFM data. The color scale can be used to compare strength of out-of-plane magnetization components quantitatively.

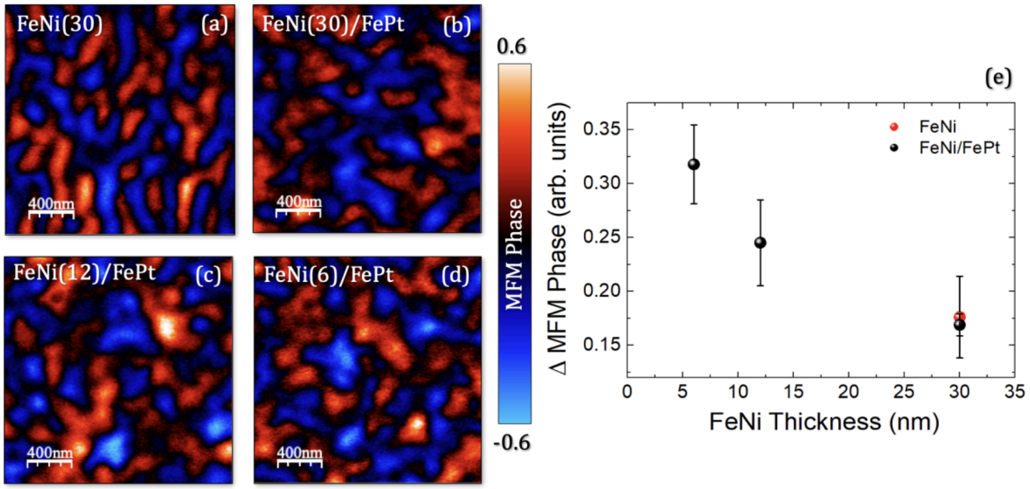

FigureS1: (a-d): MFM phase maps, 2 μm x 2 μm in lateral size, of FeNi/FePt with thickness varying from 30 to 6 nm. All maps share the same color contrast range, to favor their direct comparison. The (c) and (d) maps show higher density of bright areas, as a result of a slightly stronger out-of-plane magnetization. (e) Plot of the span between the highest and lowest MFM signal Δ, measured across 3 sampled areas per each sample, vs FeNi Thickness. Red scatter is FeNi/Si, blue scatters are FeNi/FePt.

III Magnetic measurements: The magnetic properties are measured using Vibrating Sample Magnetometer(VSM, Microsense EV-9). The hysteresis loops are recorded at room temperature

by applying the magnetic field both parallel (in-plane) and perpendicular to the film plane (out-plane). Figure S2 shows the magnetic data, which clearly indicate that as the thickness of the top FeNi layer decreases from 30 nm down to 6 nm, the easy axis of magnetization undergoes a transformation from in-plane towards out-of-plane alignment. This observed transition is further corroborated by MFM results.

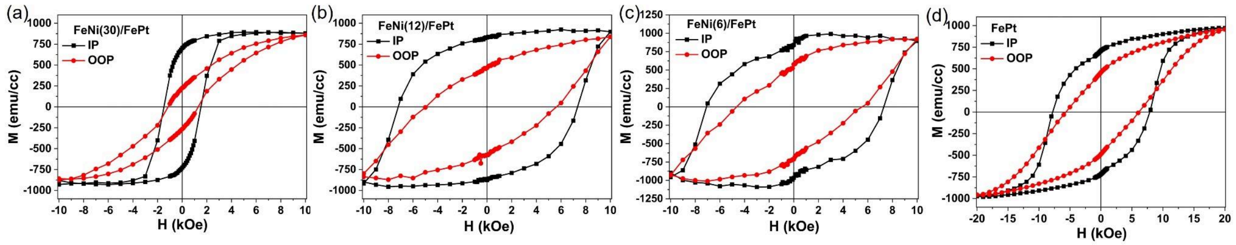

FigureS2: Magnetic hysteresis curve for (a-c) FeNi/FePt with different FeNi thickness, (d) FePt film.

IV Estimated thickness/diffusion from Rutherford back scattering (RBS) measurements: Fig.S2 shows the RBS data for FeNi(6)/FePt and FeNi(12)/FePt composite magnets. These samples show the transient magnetization enhancement on ultrafast timescales. Helium ions (0.5–4 MeV) are directed at the sample, and the energy of backscattered projectiles provides insights into film characteristics. In this study, RBS measurements were performed at IUAC, New Delhi, India, employing a 2 MeV He2+ ion beam utilizing 1.7 MV pelletron accelerator and alphatron ion source for producing He2+ ions to assess the thickness and depth profile alterations resulting from thermal annealing. The RBS spectrum, obtained experimentally, undergoes fitting with XRUMP software to obtain estimated thicknesses and diffusion in proximity of interface. The values are listed in Table S2.

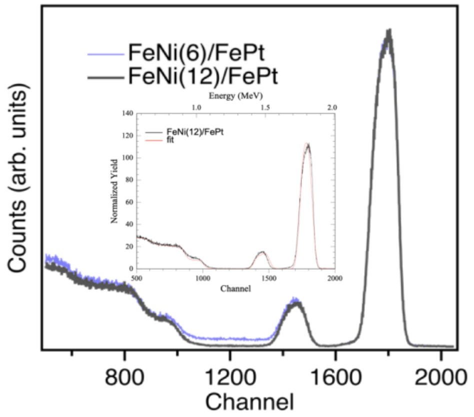

Figure S3: RBS data taken on FeNi/FePt composite samples. Inset: Fitted FeNi(12)/FePt to estimate the thickness and diffusion.

TABLE S2: Film thickness/interlayer diffusion estimated from RBS on FeNi/FePt samples.

**Sample**	**Substrate (SiO_2/Si) (nm)**	**FePt-SiO2 Diffusion (nm)**	**$Fe_{46}Pt_{54}$ (nm)**	**FePtNi (diffuse layer) (nm)**	**$Fe_{20}Ni_{80}$ (nm)**
FeNi(6)/FePt	450/bulk	25	112	9	2
FeNi(12)/FePt	450/bulk	25	102	20	7

V Estimated absorption in each layer in FeNi/FePt magnet: In terms of effective fluence, we calculated the volumetric energy density, i.e., absorption, in each layer of the sample stack in the optical region. The resulting values are presented in the table below. Notably, the exchange coupling is between two layers, thus the volumetric energy deposited in the FeNi/FePt stack is 42% for FeNi(6)/FePt and 37% for FeNi(12)/FePt. Consequently, the effective fluence in both samples is nearly similar, with a modest difference of less than 5%.

TABLE S3: Estimated absorption in each layer in the optical region.

**Sample**	**Absorption of incident laser (%)**
FeNi(6)/FePt	FeNi : 16.3 ; FePt: 26.1
FeNi(12)/FePt	FeNi: 22.1 ; FePt: 14.6
